# Supplementary material for: Vaccine effectiveness against SARS-CoV-2 infection, hospitalization, and death when combining a first dose ChAdOx1 vaccine with a subsequent mRNA vaccine in Denmark: A nationwide population-based cohort study
Source: PLoS Med. 2021 Dec 17;18(12):e1003874. doi: 10.1371/journal.pmed.1003874 (PMC8726493; doi:10.1371/journal.pmed.1003874)
Supplement: S1 Table — (DOCX) [file pmed.1003874.s002.docx]

**S1 Table. Overview of the International Classification of Diseases, 10th revision (ICD-10) codes included in the comorbidity covariate.**

| **Disease** | **ICD-10 codes** |
| --- | --- |
| Diabetes | E10-E14 |
| Adiposity | E65-E68 |
| Cancer | C00-C80, Z85 |
| Neurological diseases | G10-G14, G20-G23, G35-G37, G71-G73, G80-G83, G90-G91, G93-G96, G99, M51 (without G360, G902) |
| Kidney diseases | N180-N200Z, N18, Z992 |
| Hematological cancers | C81-C96, Z856-Z857 |
| Cardiovascular diseases | I20, I230-I259Z, I45-I499Z, I24, I50-I099Z, I340-I399Z, I05, I44, I21-I238Z, I000-I029Z, I30-I399Z, I26-I289Z, I40-I439Z, I50-I528Z, R01-R012B, I10-I59Z |
| Respiratory diseases | J400-J998Z, J40, J100-J229Z, J68, J430-J499 |
| Immune diseases | B200-B249Z, B20, Z21, D800-D899Z, Z923, Z926, Z941-Z949, Z94 (without Z945, Z947) |
| Other diseases | K700-K709, K70, E150-E909Z, D500-D649Z, D709-D779Z, D50, D650-D699Z, K710-K778Z, Q200-Q349Z, A150-A199Z, A15, Z902, Z905, Y90, Y900-Y919, Z251, |
