# Supplementary material for: Vaccine effectiveness against SARS-CoV-2 infection, hospitalization, and death when combining a first dose ChAdOx1 vaccine with a subsequent mRNA vaccine in Denmark: A nationwide population-based cohort study
Source: PLoS Med. 2021 Dec 17;18(12):e1003874. doi: 10.1371/journal.pmed.1003874 (PMC8726493; doi:10.1371/journal.pmed.1003874)
Supplement: S2 Table — (DOCX) [file pmed.1003874.s003.docx]

**S2 Table. Definition of country of origin.**

| **Country of origin** | **Definition** |
| --- | --- |
| Denmark | Individuals who were born in Denmark or abroad and have at least one parent who is Danish citizen and born in Denmark. |
| High-income country | Individuals with country of origin* of Nordic countries, EU countries, Andorra, Liechtenstein, Monaco, San Marino, Switzerland, the United Kingdom, the Vatican City, Canada, the United States, Australia and New Zealand. |
| Other country | Individuals with country of origin* of all other countries than the countries defined by western heritage. |

*Countries of origin is based on the following definitions:

- When neither parent is known, the country of origin is defined on the basis of the person's own information. If the person is an immigrant, it is assumed that the country of origin is equal to the country of birth. If the person is a descendant, it is assumed that the country of origin is equal to the country of citizenship.
- When only one parent is known, the country of origin is defined based on its country of birth. If this is Denmark, the country of citizenship is used.
- When both parents are known, the country of origin is defined on the basis of the mother's country of birth and country of citizenship, respectively.
