# Supplementary material for: Vaccine effectiveness against SARS-CoV-2 infection, hospitalization, and death when combining a first dose ChAdOx1 vaccine with a subsequent mRNA vaccine in Denmark: A nationwide population-based cohort study
Source: PLoS Med. 2021 Dec 17;18(12):e1003874. doi: 10.1371/journal.pmed.1003874 (PMC8726493; doi:10.1371/journal.pmed.1003874)
Supplement: S3 Table — (DOCX) [file pmed.1003874.s004.docx]

**S3 Table. Unadjusted and adjusted vaccine effectiveness (VE) estimates against RT-PCR SARS-CoV-2 infection of one dose of the ChAdOx1 vaccine and the ChAdOx1/BNT162b2 mRNA or ChAdOx1/mRNA-1273 vaccine schedule, respectively.**

|  |  |  |  |  | Unadjusted | | Adjusted* | |
| --- | --- | --- | --- | --- | --- | --- | --- | --- |
| Vaccine type | **Time interval (days)** | **No. of events** | **Person-years** | **Incidence rate** | **VE, %** | **95% CI** | **VE, %** | **95% CI** |
| Unvaccinated |  | 81,894 | 1,652,793 | 0.0495 | Reference |  | Reference |  |
| The ChAdOx1/ BNT162b2 mRNA vaccine schedule | 0-13 | 80 | 3,350 | 0.0239 | 52 | 28; 68 | 62 | 52; 69 |
|  | ≥14 | 23 | 5,298 | 0.0043 | 91 | 82; 96 | 87 | 80; 91 |
| The ChAdOx1/mRNA-1273 vaccine schedule | 0-13 | 29 | 1,853 | 0.0157 | 68 | 39; 84 | 73 | 62; 82 |
|  | ≥14 | 6 | 2,844 | 0.0021 | 96 | 82; 99 | 93 | 84; 97 |

*Adjusted for calendar time, age, sex, country of origin, hospital admission and comorbidity.

CI, confidence interval.
