# Supplementary material for: Vaccine effectiveness against SARS-CoV-2 infection, hospitalization, and death when combining a first dose ChAdOx1 vaccine with a subsequent mRNA vaccine in Denmark: A nationwide population-based cohort study
Source: PLoS Med. 2021 Dec 17;18(12):e1003874. doi: 10.1371/journal.pmed.1003874 (PMC8726493; doi:10.1371/journal.pmed.1003874)
Supplement: Attachment — Submitted filename: gram.pdf [file pmed.1003874.s005.pdf]

This paper examines the effectiveness of a hybrid vaccination strategy against COVID–19 in Denmark.

I was asked for a statistical report and I interpret that to include all aspects of the design and conduct of the study.

Points of detail

Page 6 Classifying ethnicity or race is an area where different countries all seems to choose idiosyncratic systems. It would be impossible to map the one outlined in S2 to the system used in the UK for various reasons. Is this the standard Danish official classification?

Page 7 Did the authors deal with overdispersion by fitting a quasi–Poisson model (using `family=quasiposisson` in R)? I believe there are other ways. Perhaps clarify?

Page 8 'However, the VE estimates were not significant ...'. That is not the interpretation I would draw from Figure 2. What Figure 2 shows is that the estimates are broadly similar throughout. The reason later time points fail to meet some arbitrary level of statistical significance is the increase in variability presumably related to the number of people remaining at risk at those time points.

Page 13 '... second dose of an mRNA vaccine is needed to maintain ...'. In order to justify that statement the authors would need to show that a second dose of some other vaccine would not have been effective and I do not think they have presented such an analysis. The word needed seems particularly inappropriate.

Figure 1 It might be helpful to additionally label the variants with the WHO names as is done in the text on page 8.

Colloquially in my country ChAdOx1 is typically called the Oxford/AstraZeneca vaccine so to aid search engine optimisation the authors might want to include that somewhere. We do not use the trade names (my GP records do not contain Cominarty).

For similar reasons I would also suggest putting Denmark or Danish in the title.

Points of more substance

Who was included in the analysis?

It is not totally clear to me what happened in Denmark at the relevant time. We seem to have three groups of people involved here (a) one dose of ChAdOx1, (b) one dose of ChAdOx1 followed by a dose of either of the mRNA vaccines, (c) unvaccinated people.

- I assume that group (a) is not a subset of (b), that is to say that group (a) for some reason did not receive any further doses at all. If so, why? If not so, then what implications does that have for the inferences.
- It also seems that nobody in Denmark at the relevant time was on a schedule which involved two doses of an mRNA vaccine so that groups (a), (b) and (c) are together exhaustive of the 5,542,079 people in the study. Is that true? Figure 1 seems to suggest the contrary.

This could all usefully be clarified and the implications discussed.

Choice of comparator

In the discussion the authors make narrative comparisons against studies which have investigated a different strategy, two doses of mRNA. I do not think more than a handful of healthcare professionals believes that any of the vaccines is ineffective, the question is whether any of them is more effective than another. So, is comparing against the unvaccinated as the authors have done appropriate? It seems that a comparison directly with the two mRNA strategy would have been possible but it is not presented.

There must be a good reason why this was not done but it is not obvious to me. The authors need to bear in mind that in an open access journal non–vaccinologists may be reading and the nuances of vaccine studies may not be apparent to them.

Details of the Cox model

In the Cox regression for the vaccinated presumably time starts when they are given the relevant dose but when does the clock start for the unvaccinated? Was the assumption of proportionality of hazards tested formally or graphically?

Summary

As can be seen I am confused about what exactly went on here.

Michael Dewey
