# Supplementary material for: Vaccine effectiveness against SARS-CoV-2 infection, hospitalization, and death when combining a first dose ChAdOx1 vaccine with a subsequent mRNA vaccine in Denmark: A nationwide population-based cohort study
Source: PLoS Med. 2021 Dec 17;18(12):e1003874. doi: 10.1371/journal.pmed.1003874 (PMC8726493; doi:10.1371/journal.pmed.1003874)
Supplement: Attachment — Submitted filename: Requests.docx [file pmed.1003874.s006.docx]

Dear editors and reviewers,

Thank you for taking your time to comment on the manuscript. Your comments have been very helpful to improve the manuscript. We have added supporting details to the methods to clarify the misunderstandings of the study design. Furthermore, a sensitivity analyses has been added. The information have been added in the manuscript and a point by-point response is included below.

# Comments from the Academic Editor:

There is a lot unclear about the methods and statistical analysis of the study. Who was included, what was the vaccination status of the rest of the population, were there people with 2 mRNA vaccinations, would it make sense to compare these to the ones included in the study? Also, the statistical analysis is described only very briefly, without any justification for underlying assumptions. How do the estimated HRs and incidences compare to the observed incidence at the time of observation? I am sure that the authors have a very good and complete data set, so it would be worthwhile extracting more complete information, adding new analyses and substantially improving presentation of the methods.

**Response:**

Thank you for your comments. We acknowledge that the description of the study design needs more clarification. Especially about the exposure variable, which is included as a time varying covariate. We have added more information in the method section and added a flow chart of the study population. We hope this will contribute to a better understanding of the study design. As described in the method section, all individuals who were residents in Denmark on 9^th^ of February 2021 or immigrated before the end of study on 23^rd^ of June 2021 were included in the study population. Only individuals with a SARS-CoV-2 infection before start of study or individuals who had been vaccinated before start of study were excluded. We included 97.6% of the total Danish population. Thereby, all study participants were unvaccinated at start of study on 9^th^ of February 2021. The study participants were included in the exposure group if they received the first dose of the ChAdOx1 or/and the ChAdOx1/mRNA vaccine schedule during the study period. However, they were included as unvaccinated until the day they received the first vaccine dose. The individuals who received an mRNA vaccine as the first dose during the study period contributed with follow-up time until the day they received the mRNA vaccine and after that, they were censored. This means that only those who received the ChAdOx1 vaccine as the first dose and the mRNA as the second dose were compared with the unvaccinated. A flow chart has been added to illustrate this (Fig 1).

# Requests from the editors:

**General comments:**

Throughout the paper, please adapt reference call-outs to the following style: "... low blood platelet counts [3,4]." (noting the absence of spaces within the square brackets).

Response: This has been corrected as requested.

**Data availability:**

PLOS Medicine requires that the de-identified data underlying the specific results in a published article be made available, without restrictions on access, in a public repository or as Supporting Information at the time of article publication, provided it is legal and ethical to do so. If the data are not freely available, please describe briefly the ethical, legal, or contractual restriction that prevents you from sharing it. Please state the owner of the data set and contact information for data requests (web or email address). Note that a study author cannot be the contact person for the data.

Response: The information has been added to the method section page 9, line 8-11: “The data included in this research are part of the Danish national COVID-19 surveillance system database at Statens Serum Institut. The data are available for research upon reasonable request and with permission from the Danish Data Protection Agency and the Danish Health Data Authority:

<https://sundhedsdatastyrelsen.dk/da/english/health_data_and_registers/research_services>”.

**Title:**

Please revise your title according to PLOS Medicine's style. Please place the study design in the subtitle (ie, after a colon). We suggest “Vaccine effectiveness against SARS-CoV-2 infection, hospitalization and death when combining a first-dose ChAdOx1 vaccine with a subsequent mRNA vaccine in Denmark: a nationwide population-based cohort study” or similar.

Response: The title has been changed in the manuscript as requested.

**Abstract:**

Please structure your abstract using the PLOS Medicine headings (Background, Methods and Findings, Conclusions).

Response: The headings have been changed in the manuscript as requested.

Abstract Background: Please expand upon the context of why the study is important. The final sentence should clearly state the study question.

Response: The information has been added as requested, page 2, line 3-6: The aim of this Danish nationwide population-based cohort study was therefore to estimate the VE against SARS-CoV-2 infection and COVID-19 related hospitalization and death following the first dose of the ChAdOx1 vaccine and the combination of the ChAdOx1/mRNA vaccines.

Abstract Methods and Findings:

Please provide brief demographic details of the study population (e.g. sex, age, ethnicity, etc).

Response: The information has been added as requested, page 2, line 18-20: The study population had a median age of 45 years when receiving the first ChAdOx1 vaccine dose, males and females were equally distributed and the majority had Danish heritage.

Please include the dates during which the study took place and length of follow up.

Response: The information has been added as requested, page 2, line 8-9: All individuals alive in or immigrating to Denmark from the 9th of February 2021 to the 23rd of June 2021 were identified in the Danish Civil Registration System and page 2, line 17-18: . A total of 1,691,464 person-years and 83,034 SARS-CoV-2 infections were included.

In the last sentence of the Abstract Methods and Findings section, please describe 2-3 of the main limitations of the study's methodology.

Response: The information has been added as requested, page 3, line 1-3: Unmeasured confounders and an increasing overall vaccine coverage in the general population creating herd immunity are important to take into consideration when interpreting the results.

**Abstract Conclusions:**

Please begin your Abstract Conclusions with "In this study, we observed ..." or similar, to summarize the main findings from your study without overstating your conclusions.

Response: The sentence has been corrected in the manuscript, page 3, line 3-5: In this study, we observed, a high reduction in the risk of SARS-CoV-2 infection when combining the ChAdOx1 and an mRNA vaccine, compared with unvaccinated individuals.

**Author Summary:**

At this stage, we ask that you include a short, non-technical Author Summary of your research to make findings accessible to a wide audience that includes both scientists and non-scientists. The Author Summary should immediately follow the Abstract in your revised manuscript. This text is subject to editorial change and should be distinct from the scientific abstract. Please see our author guidelines for more information: <https://journals.plos.org/plosmedicine/s/revising-your-manuscript#loc-author-summary>. In the final bullet point of ‘What Do These Findings Mean?’, please describe the main limitations of the study in non-technical language.

Response: The information has been added as requested.

**Introduction:**

Line 66 - please temper assertions of primacy by adding ‘to the best of our knowledge’ or similar.

Response: The sentence has been rephrased from: However, no previous studies have reported VE of a ChAdOx1/mRNA vaccine schedule. To: However, to the best of our knowledge no previous studies have reported VE of a ChAdOx1/mRNA vaccine schedule, page 5, line 6-7.

**Methods:**

Did your study have a prospective protocol or analysis plan? Please state this (either way) early in the Methods section. If a prospective analysis plan (from your funding proposal, IRB or other ethics committee submission, study protocol, or other planning document written before analyzing the data) was used in designing the study, please include the relevant prospectively written document with your revised manuscript as a Supporting Information file to be published alongside your study, and cite it in the Methods section. A legend for this file should be included at the end of your manuscript. If no such document exists, please make sure that the Methods section transparently describes when analyses were planned, and if/when reported analyses differed from those that were planned. Changes in the analysis-- including those made in response to peer review comments-- should be identified as such in the Methods section of the paper, with rationale. If a reported analysis was performed based on an interesting but unanticipated pattern in the data, please be clear that the analysis was data-driven.

Response: There is no prospective study protocol for the study. However, we have added some more information to the method section to transparently describe the analyses**.**

Please ensure that the study is reported according to the RECORD guideline, and include the completed RECORD checklist as Supporting Information. Please add the following statement, or similar, to the Methods: "This study is reported as per the REporting of studies Conducted using Observational Routinely-collected health Data (RECORD) Statement (S1 Checklist)." The RECORD guideline can be found here: https://www.equator-network.org/reporting-guidelines/record/ When completing the checklist, please use section and paragraph numbers, rather than page numbers which will likely no longer correspond to the appropriate sections after copy-editing.

Response: The statement has been added to the method section, page 6, line 13-14. The RECORD checklist has been added to the supporting information.

Please provide an ethics statement in your Methods section.

Response: An ethic statement has been added to the methods section with following heading: Ethical considerations. Following statement has been added, page 9, line 7: The present study was based on existing administrative data and did not require ethical approval.

**Results:**

Line 143 - please provide a reference to Table S1 in which details of comorbidities are provided, and provide a few examples of these comorbidities in the main text.

Response: The information has been added as requested: In the study population, 25.6% had at least one of the comorbidities defined in Table S2, with the comorbidity category other diseases, cardiovascular diseases and respiratory diseases being most prevalent.

Lines 156-178 - please clarify what is meant by ' Significant adjusted VE estimates’ and ‘VE estimates were not significant’.

Response: Statistically significant is meant by ‘significant’. The section has been changed to, page 11, line 1-5: The adjusted VE estimates against SARS-CoV-2 infection between 14-83 days after one dose of the ChAdOx1 vaccine were relatively stable with overlapping CIs. These VE estimates ranged from 29% (95% CI: 12; 43) to 44% (95% CI: 32; 55) after one dose of the ChAdOx1 vaccine. The VE estimates were not statically significant at 0-13 days, 42-48 days and from 84 days. However, this may be explained by the number of people remaining at risk at those time points.

**Discussion:**

Please present and organize the Discussion as follows: a short, clear summary of the article's findings; what the study adds to existing research and where and why the results may differ from previous research; strengths and limitations of the study; implications and next steps for research, clinical practice, and/or public policy; one-paragraph conclusion. Please remove all subheadings within your Discussion e.g. Strengths and limitations.

Response: The discussion has been reorganized and the subheading has been removed as requested.

**Figures:**

Please provide titles and legends for all figures (including those in Supporting Information files).

Response: The information has been added as requested.

Please indicate in the figure caption the meaning of the bars [and whiskers] in Figure 2.

Response: The information has been added as requested.

**Tables:**

Please present numerators and denominators in your tables.

Response: The information has been added in the tables as requested.

Please specify the variables controlled for in Tables 2, 3 and 4.

Response: The variables controlled for in Table 2, 3 and 4 is already described under the table and referred to as*.

**References:**

Please ensure that journal name abbreviations match those found in the National Center for Biotechnology Information (NCBI) databases, and are appropriately formatted and capitalised.

Please also see https://journals.plos.org/plosmedicine/s/submission-guidelines#loc-references for further details on reference formatting.

Response: The references have been corrected as requested.

**Supplementary files:**

Please provide titles and legends for each individual table in the Supporting Information.

Response: The information has been added as requested.

# Comments from the reviewers:

**Reviewer #1:**

This is a very nice and important study that utilizes the power of the Danish health informatics system, and the decision to suspend the use of ChAdOx1, to assess the efficacy of the ChAdOx1/mRNA combination.

The results are important and valuable for global vaccine policy.

**Points**

- two different mRNA vaccines were used. Is there sufficient statistical power to assess if there is any difference between these? They have different doses and there is a suggestion that Moderna may be more potent.

Response: Thank you for your this suggestion, we examined it and have added the analysis to the manuscript.

- the time interval between first and second dose if important for immune response. This appears to vary and I wonder if a plot of 'time between doses', with median and range, may be a useful addition?

Response: We agree that this information is useful. The information is available in the last row of Table 1.

- very minor corrections of English - eg use of double negative in last sentence.

Response: Thank you. We have read through the manuscript again and corrected where applicable.

**Reviewer #2**:

This paper describes the effectiveness of a single dose of ChAdOx1 vaccination as well as ChAdOx1 followed by a mRNA vaccination. The findings from this study are important as countries are considering differing vaccination schedules and ways to conserve and share doses. Strengths of the study include its population based, large sample size, and near-complete capture of key data on infections, healthcare utilization, and vaccinations.

**Specific comments for the authors:**

Background - lines 56-57: "due to the present epidemiological situation" - not sure what this means? Because of the blood clot data - or because of patterns of SARS-CoV-2 circulation**?**

Response: Sorry for the confusion. Yes, this is because of patterns of SARS-CoV-2 circulation and has been clarified in the text, page 4, line 17-19.

**Assessment of exposure - lines 89-91:**

Individuals who exclusively received one or two doses of mRNA vaccines were excluded from analysis, correct?

Response: Apologies if the study design was unclear. We have added more details and a flow chart to clarify this misunderstanding. Exclusions from the analysis were as follows: individuals who received an mRNA vaccine before start of study were excluded. Individuals who received an mRNA vaccine as the first dose were censored when they received the first mRNA vaccine, meaning that they were followed as unvaccinated individuals until they received an mRNA vaccine as the first dose.

Not sure what is meant by "western" and "non-western" heritage. Please explain further.

Response. We have used the official definition from Statistic Denmark. Additional information has been added in Table S3: Definition of heritage to help future readers.

**Results - lines 138-140:**

Is it correct that 7,809 individuals received one ChAdOx1 vaccine but did not go on to receive a second dose of any product? Or did some of these individuals receive a second dose of ChAdOx1? Were these individuals excluded from the analysis, or did they contribute to the one ChAdOx1 dose VE estimate? How many people in the population were unvaccinated or exclusively received mRNA vaccines? I think it's important to report the size of the unvaccinated sample since they are used as the comparison group.

Response: Thank you for pointing out the need to clarify. We acknowledge that the description of the study design needs more clarification. Especially about the exposure variable, which is included as a time varying covariate. We have added more information in the method section and added a flow chart of the study population. It is correct that we included 7,809 individuals who received the first dose of the ChAdOx1 vaccine but not in the combined vaccine schedule. The reason is that some individuals are censored between receiving the first or second dose due to SARS-CoV-2 infection (outcome), death, emigration or vaccination with a second dose of the ChAdOx1 vaccine.

**Line 168: typographical error:**

"receptively" should be "respectively".

Response: Thank you. This has been corrected in the manuscript.

**All-cause mortality**:

This seems a little problematic as an outcome in this VE analysis because it is a very non-specific outcome. Could the authors provide a little more explanation about why they chose to investigate this outcome? Did the authors believe that COVID-related deaths would be missed with their case definition (even though they report that testing was common in Denmark)? All-cause hospitalization also seems like a non-specific outcome.

Response: Thank you for your comment. We agree that all-cause hospitalization and death are non-specific outcomes. We have therefore decided to remove the results concerning these outcomes. The reason why we choose to investigate these outcomes in the first place was due to few cases of COVID-19 related hospitalization and death within the study population. However, we think the message of this manuscript is more clear without these outcomes.

COVID-19 hospitalizations and deaths based on positive PCR results prior to events - the authors note this as a limitation. Could diagnosis codes or cause of death have been incorporated to improve the specificity of the case definition?

Response: We agree that this could be relevant. However, our definitions follows international guidelines from the European Centre for Disease Prevention and Control (ECDC). Furthermore, the ICD-10 codes for COVID-19 may not me registered if the person is admitted with underlying diseases. Although we mention this definition as a limitation, we expect that the possible misclassification is non-differential.

In Table 1, it appears the authors are able to distinguish front-line personnel from other vaccine recipients. The authors note that front-line personnel may have different SARS-CoV-2 exposures and PPE use behaviors that might have impacted VE. Did the authors consider a sensitivity analysis limiting to front-line workers to see if that impacted their findings?

Response: We are able to distinguish vaccinated front-line personnel but we are not able to distinguish unvaccinated front-line personnel. Therefore, it is unfortunately not possible to add this information in a sensitivity analysis.

**Table 1** - "median number of days to the first dose" - not sure what this means? Wasn't follow up time started at the date of the first dose?

Response: Thank you very much for your question. Follow-up started for all individuals on the 9^th^ of February 2021.

**Reviewer #3**

**Points of detail**

Page 6 Classifying ethnicity or race is an area where different countries all seems to choose idiosyncratic systems. It would be impossible to map the one outlined in S2 to the system used in the UK for various reasons. Is this the standard Danish official classification?

Response: Yes, this is an official classification from Statistics Denmark. We have added additional information on the definition of heritage in Table S3.

Page 7 Did the authors deal with overdispersion by fitting a quasi{Poisson model (using family=quasiposisson in R)? I believe there are otherways. Perhaps clarify?.

Response: Yes. We have now specified this in the method section, page 8, line 13**.**

Page 8 `However, the VE estimates were not significant . . . '. That is not the interpretation I would draw from Figure 2. What Figure 2 shows is that the estimates are broadly similar throughout. The reason later time points fail to meet some arbitrary level of statistical significance is the increase in variability presumably related to the number of people remaining at risk at those time points.

Response: Thank you for…. We have modified the interpretation according to the reviewer’s suggestion and now write: The VE estimates were not statically significant at 0-13 days, 42-48 days and from 84 days. However, this may be explained by the number of people remaining at risk at those time points (page 11, line 4-5).

Page 13 `. . . second dose of an mRNA vaccine is needed to maintain . . . '. In order to justify that statement the authors would need to show that a second dose of some other vaccine would not have been effective and I do not think they have presented such an analysis. The word needed seems particularly inappropriate. Response: The interpretation has been changed as suggested.

Figure 1 It might be helpful to additionally label the variants with the WHO names as is done in the text on page 8.

Response: The WHO names has been added as suggested.

Colloquially in my country ChAdOx1 is typically called the Oxford/AstraZeneca vaccine so to aid search engine optimisation the authors might want to include that somewhere. We do not use the trade names (my GP records do not contain Cominarty).

Response: It is already defined in the introduction that ChAdOx1 is the vaccine from AstraZeneca. Oxford have been added as suggested.

For similar reasons I would also suggest putting Denmark or Danish in the title.

Response: Thank you for your suggestion. We have now added this information to the title.

**Points of more substance**

Who was included in the analysis? It is not totally clear to me what happened in Denmark at the relevant time. We seem to have three groups of people involved here (a) one dose of ChAdOx1, (b) one dose of ChAdOx1 followed by a dose of either of the mRNA vaccines, (c) unvaccinated people.

- I assume that group (a) is not a subset of (b), that is to say that group (a) for some reason did not receive any further doses at all. If so, why? If not so, then what implications does that have for the inferences.
- It also seems that nobody in Denmark at the relevant time was on a schedule which involved two doses of an mRNA vaccine so that groups (a), (b) and (c) are together exhaustive of the 5,542,079 people in the study. Is that true? Figure 1 seems to suggest the contrary.

This could all usefully be clarified and the implications discussed.

Response: We have added supporting information to the method section to clarify the misunderstandings of the study design. A total of 5,542,079 individuals were included in the analysis (97.6% of the total Danish population). Individuals were excluded if they had received a COVID-19 vaccine or have had a SARS-CoV-2 infection before start of study. All individuals were unvaccinated at start of study (9^th^ of February 2021). This means that the study did not have three groups of individuals at start of study but one group of unvaccinated individuals. The exposure variable was included as a time-varying variable. The individuals are followed as unvaccinated until the first dose of the ChAdOx1 vaccine (exposed), the first dose of another COVID-19 vaccine (censored), death (censored), emigration (censored), SARS-CoV-2 infection (outcome) or end of follow-up. As shown in Figure 1, the majority of individuals received an mRNA vaccine as the first and second dose.

**Choice of comparator**

In the discussion the authors make narrative comparisons against studies which have investigated a different strategy, two doses of mRNA. I do not think more than a handful of healthcare professionals believes that any of the vaccines is ineffective, the question is whether any of them is more effective than another. So, is comparing against the unvaccinated as the authors have done appropriate? It seems that a comparison directly with the two mRNA strategy would have been possible but it is not presented.

There must be a good reason why this was not done but it is not obvious to me. The authors need to bear in mind that in an open access journal non{vaccinologists may be reading and the nuances of vaccine studies may not be apparent to them.

Response: Unvaccinated individuals comprised the reference group, according to the definition from Centers for Disease Control and Prevention (CDC), that is “*Vaccine effectiveness (VE) is measured by calculating the risk of disease among vaccinated and unvaccinated persons and determining the percentage reduction in risk of disease among vaccinated persons relative to unvaccinated persons*”^[1]^.

Details of the Cox model In the Cox regression for the vaccinated presumably time starts when they are given the relevant dose but when does the clock start for the unvaccinated?

Response: The time started on the 9^th^ of February 2021 for all individuals. All included individuals were unvaccinated at start of study (9^th^ of February 2021). The exposure variable was included as a time-varying variable. The individuals were followed as unvaccinated until the first dose of the ChAdOx1 vaccine (exposed), the first dose of another COVID-19 vaccine (censored), death (censored), emigration (censored), SARS-CoV-2 infection (outcome) or end of follow-up, whichever event occurred first. We have added more information in the method section and added a flow chart of the study population. We hope this will contribute to a better understanding of the study design.

Was the assumption of proportionality of hazards tested formally or graphically?

Response: The proportionality of hazards were tested graphically. The information has been added to the statistical analysis section, page 8, line 18-19).

1. <https://www.cdc.gov/csels/dsepd/ss1978/lesson3/section6.html>
