# Supplementary material for: Vaccine effectiveness against SARS-CoV-2 infection, hospitalization, and death when combining a first dose ChAdOx1 vaccine with a subsequent mRNA vaccine in Denmark: A nationwide population-based cohort study
Source: PLoS Med. 2021 Dec 17;18(12):e1003874. doi: 10.1371/journal.pmed.1003874 (PMC8726493; doi:10.1371/journal.pmed.1003874)
Supplement: Attachment — Submitted filename: Response to Editors.docx [file pmed.1003874.s007.docx]

**Requests from Editors:**

At line 37 and any other instances in the paper, please rephrase "heritage", "Western" and "non-Western" to avoid potential stigmatization. Are you able to substitute "Danes", "European migrants", "non-European migrants" and "others", for example?

Response: Thank you very much, for your help to decide on the optimal language to use. After further discussion with Richard Turner, we decided to rephrase heritage: Country of origin (Denmark, high-income countries, other countries).

At line 41, please adapt the final sentence of the "Methods and findings" subsection of your abstract to begin "Study limitations include ..." or similar.

Response: Has been added.

At line 43, please remove the comma after "observed" and make that "large" rather than "high" (reduction).

Response: Has been added.

At line 58 (author summary) please make that "... associated with estimated protection ...".

Response: Has been added.

At line 68, please use the notation "... 4.1 million".
Response: Has been corrected.

Please state explicitly in the Methods section (main text) that "The study did not have a prespecified analysis plan." or similar, assuming this is the case.

Response: Has been added.

Please remove the statement on Data Availability from the Methods section (main text). In the event of publication, this information will appear in the article metadata, via entries in the submission form.

Response: Has been removed.

Throughout the text, please style reference call-outs as follows: "... platelet counts [3,4]." (noting the absence of spaces within the square brackets).

Has been corrected.

For reference 5 and other citations to the journal, "Lancet" will suffice as the journal name.

Has been corrected.

Please add "[Preprint]" to reference 6 and any other preprints that are cited, unless you are able to substitute the corresponding peer-reviewed publications.

Response: Has been added.

Noting reference 7, please ensure that all references have full access details.

Has been corrected.

Thank you for including a completed RECORD checklist. Please break this out into a separate attachment, labelled "S1_RECORD_Checklist" or similar and referred to as such in your Methods section (main text).

Has been added.
